# One-Step Lithium Bioleaching from a Mineral Concentrate: Comparison Between Consortium and Isolated Native Strains

**DOI:** 10.3390/ma19132855

**Published:** 2026-07-03

**Authors:** María Guadalupe Quezada-Aldaco, Gloria Abigail Martinez-Rodriguez, Juan Antonio Rojas-Contreras, Perla Guadalupe Vázquez-Ortega, Hiram Medrano-Roldán, Damián Reyes-Jáquez, Norma Urtiz-Estrada, Karla Torres-Fraga, David Enrique Zazueta-Álvarez, Grisel Fierros-Romero, Alma Karina Tamez-Castrellón

**Affiliations:** 1 Departamento de Ingenierías Química y Bioquímica, TecNM/Instituto Tecnológico de Durango, Blvd. Felipe Pescador 1830 Ote., Durango 34080, Mexico; 22040317@itdurango.edu.mx (M.G.Q.-A.); 22040319@itdurango.edu.mx (G.A.M.-R.); jrojas@itdurango.edu.mx (J.A.R.-C.); pvazquez@itdurango.edu.mx (P.G.V.-O.); hiramdurango@yahoo.com.mx (H.M.-R.); 2 Facultad de Ciencias Químicas, Universidad Juárez del Estado de Durango, Av. Veterinaria s/n. Circuito Universitario, Col. Valle del Sur, Durango 34120, Mexico; norma.urtiz@ujed.mx; 3 Departamento de Ingeniería en Tecnología Ambiental, Universidad Politécnica de Durango, Carretera Durango-México Km. 9.5, Durango 34300, Mexico; karla.torres@unipolidgo.edu.mx; 4 TecNM/Instituto Tecnológico Superior de Ciudad Hidalgo, Av. Ing. Carlos Rojas Gutierrez 2120, Fracc. Valle de la Herradura, Ciudad Hidalgo 61100, Mexico; griselfierrosromero@gmail.com; 5 Unidad de Posgrado e Investigación y Desarrollo Tecnológico, Instituto Tecnológico de Durango, Blvd. Felipe Pescador 1830 Ote., Col. Nueva Vizcaya., Durango 34080, Mexico; soulk_taca@hotmail.com

**Keywords:** bioleaching, native microorganisms, mineral extraction

## Abstract

**Highlights:**

- Native microbial consortia enabled one-step lithium bioleaching from mineral concentrates.
- Maximum lithium recovery (99%) was achieved at pH 8, 10% pulp, and no agitation.
- Microbial consortia outperformed individual bacterial strains in lithium solubilization.
- Mineralogical changes confirmed bioleaching feasibility for sustainable lithium extraction.

**Abstract:**

The increasing global demand for energy has intensified the need for lithium, a critical component in rechargeable batteries and electric vehicles. However, conventional lithium extraction methods are associated with significant environmental impacts. In this study, a one-step bioleaching approach for lithium recovery from mineral concentrates was evaluated using native microbial consortia and isolated bacterial strains. A suitable culture medium was selected, individual strains were isolated, and bioleaching experiments were conducted using a Box–Behnken experimental design. Lithium solubilization and cell density were assessed under different agitation conditions, pulp concentrations, and initial pH values. The highest lithium solubilization (99%) was achieved under non-agitated conditions, with a pulp concentration of 30% and an initial pH of 6. Three bacterial strains (ITDB101, ITDR102, and ITDN103) were identified. The native microbial consortium and the biotic control exhibited the highest lithium solubilization efficiencies (94.5% and 96.3%, respectively), outperforming the individual strains, which achieved solubilization values ranging from 91.73% to 93.27%. X-ray diffraction analysis identified five mineral phases in the concentrate, and comparisons among treatments revealed changes in these phases following bioleaching, supporting the potential of this process as an environmentally friendly alternative for lithium extraction.

## 1. Introduction

Lithium is a highly reactive element and is only found in nature as part of mineral compounds such as spodumene, lepidolite, and petalite [1]. It is a silvery-white metal with a low density (0.531 g/cm^3^) and an atomic weight of 6.941. Among alkali metals, lithium has the highest melting point (186 °C) and boiling point (1336 °C), as well as a high energy density of 0.784 cal/g°C at 0 °C [2]. These properties make lithium essential for a range of technological applications, including high-energy-density rechargeable batteries [3], aluminium–lithium alloys in the aerospace industry [1], and pharmaceutical applications, particularly for the treatment and maintenance of bipolar disorder [4]. Recent projections indicate that global lithium demand may exceed supply by 2030, driven by the rapid expansion of battery technologies and increasingly ambitious decarbonization targets [5].

Lithium is widely distributed in nature and occurs in igneous, volcanic, and sedimentary rocks at concentrations ranging from 20 to 70 ppm, with an average abundance of approximately 65 ppm in the Earth’s crust. It is also present in oceans, thermal fluids, and brines, where concentrations can reach up to 1000 ppm. Although lithium has been identified in more than 140 minerals, only a limited number contain concentrations that are economically viable for extraction [6]. In mineral deposits, lithium is commonly associated with magnesium, potassium, sodium, and boron [7]. Globally, lithium resources are classified into three main categories: brine-type, hard rock-type, and clay-type deposits. Among these, the primary commercial sources are natural brines formed through intense evaporation and hard rock deposits rich in lithium-bearing minerals such as lithium-cesium-tantalum pegmatites [8]. Brine-type resources represent approximately 65% of global lithium reserves and are mainly distributed within the “Lithium Triangle” [9]. The increasing demand for lithium has intensified traditional mining activities, which often lead to adverse environmental impacts. These include water contamination from toxic discharges and the emission of volatile pollutants that can affect surrounding communities [10]. In response, the mining industry has increasingly adopted environmentally sustainable alternatives such as mineral biotechnology, which uses microorganisms or their metabolites to recover metals from ores, concentrates, rocks, and solutions [11].

One promising technique within mineral biotechnology is bioleaching. This method employs microorganisms to recover metals from low-grade ores, often increasing efficiency while reducing processing time and environmental impact [12,13]. Compared to conventional pyro- and hydrometallurgical methods, such as smelting, roasting, leaching, and oxidation processes, bioleaching has demonstrated significant potential for metal recovery due to its lower energy and chemical requirements, reduced capital costs, simpler processing, and lower environmental impact [14,15].

Bioleaching microorganisms are classified according to their metabolic pathways (chemotrophs, chemoorganotrophs) and nutrient requirements. Some use mineral dissolution products as energy sources, while chemolithotrophs grow in inorganic media, oxidizing iron and sulfur compounds [13,16]. Bioleaching relies on microbial metabolic products, including organic acids, inorganic acids, and iron oxides, to extract metals from, while acidophilic and heterotrophic microorganisms contribute by secreting organic acids that form metal complexes and enhance solubilization [17]. In process design, critical factors include the selection of suitable microorganisms, often native strains, that are already adapted to survive and thrive in toxic or extreme conditions. Mixed cultures can outperform pure cultures due to synergistic interactions established among different microorganisms [18]. Recent studies have further highlighted microbial consortia as promising systems for enhancing bioleaching efficiency and metal recovery through complementary microbial interactions [19]. These microorganisms may exist in free-floating (planktonic) cells or surface-adhered (biofilm) forms. Biofilms help bacteria attach to solid substrates and resist environmental stress by producing extracellular polymeric substances (EPS) such as polysaccharides, proteins, and lipids [20,21].

Microorganisms such as *Acidithiobacillus ferrooxidans* have been used for the extraction of lithium from lepidolite and jadarite, reaching concentrations of approximately 12 mg/L in 30 days and more than 120 mg/L in 21 days, respectively. Throughout the experiment, a sustained release of lithium was observed compared to the abiotic control [22].

Meanwhile, a consortium consisting of autotrophic bacteria (*A. ferrooxidans* and *A. thiooxidans*), the heterotrophic fungus *Aspergillus niger*, and the yeast *Rhodotorula mucilaginosa* has been evaluated for the bioleaching of lithium from lepidolite. Under nutrient-limited conditions, the bacterial consortium proved to be the most effective, with a recovery of 11 mg/L of lithium after 336 days. The bioleaching percentages obtained were 8.8% for the bacterial consortium, 0.2% for *A. niger*, and 1.1% for *R. mucilaginosa* [23].

Finally, lithium bioleaching from spodumene has been applied using native heterotrophic microorganisms such as *Penicillium purpurogenum*, *Aspergillus niger*, and *Rhodotorula rubra*. In 30 days, *P. purpurogenum* bioaccumulated 10.8 mg of lithium per gram of dry weight (mg/g dw) and solubilized 1.26 ppm of lithium. *R. rubra* showed the highest bioaccumulation capacity, with 16.7 mg/g dw and solubilization of 1.53 ppm, while *A. niger* retained 5.1 mg/g dw, with 0.75 ppm of lithium found in the bioleaching liquor [24]. These findings demonstrate the potential of microorganisms for lithium recovery from minerals, both individually and through their synergistic action in a consortium.

One-step bioleaching is a process in which microorganisms and the mineral substrate are inoculated simultaneously into the culture medium, allowing microbial growth and mineral dissolution to occur concurrently. Although this approach may require a longer processing time than conventional multi-step strategies, it offers the advantage of reducing operational complexity and overall processing costs [16]. Despite these advances, few studies have directly compared the bioleaching performance of native microbial consortia and bacterial strains isolated from the same mineral source under identical experimental conditions. Such comparisons are essential for understanding the contribution of microbial interactions to lithium recovery and for selecting efficient bioleaching strategies. The present study compares the performance of a native microbial consortium and bacterial strains isolated from the same mineral concentrate in a one-step biohydrometallurgical process for lithium recovery. To this end, the bioleaching performance of the enriched consortium and the isolated bacterial strains was evaluated under identical experimental conditions using a Box–Behnken response surface design. The results provide insight into the potential of native microbial communities and individual bacterial isolates for lithium recovery through a single-stage bioleaching process.

## 2. Materials and Methods

### 2.1. Sample

The lithium-bearing mineral sample consisted of a mineral concentrate provided by Bacanora Minerals (Calgary, AB, Canada) from the “Lithium Project” located in the state of Sonora, in northeastern Mexico, approximately 11 km south of Bacadehuachi, 180 km northeast of Hermosillo, and approximately 170 km south of the US-Mexico border. The mineral concentrate had a particle size of 50 mesh and was derived from the clay mineral species polylithionite [KLi_2_Al(Si_4_O_10_)(F, OH)_2_] [25].

### 2.2. Preparation of the Microbial Enrichment System

Two culture media were evaluated to determine the most suitable conditions for the development of the native microbial consortium: 9K medium, designed for acidic environments, and API sulfate medium, formulated for alkaline conditions. The 9K medium, prepared according to Silverman et al. [26], consisted of (NH_4_)_2_SO_4_, KCl, K_2_HPO_4_, MgSO_4_·7H_2_O, Ca(NO_3_)_2_, and FeSO_4_·7H_2_O, with ferrous sulfate sterilized separately at 121 °C for 15 min. Meanwhile, the API sulfate medium, based on Vargas Rubio et al. [27], consisted of C_6_H_8_O_6_, NaCl, K_2_HPO_4_, MgSO_4_, (NH_4_)_2_Fe(SO_4_)_6_·H_2_O, and sodium lactate (NaC_3_H_5_O_3_). All salts were dissolved in distilled water and heated under constant stirring to prevent precipitation, and the complete medium was sterilized in an autoclave at 121 °C for 15 min. Both media were prepared according to their original formulations without pH adjustment. The experimental treatments with each medium were performed in triplicate at 30 °C and 180 rpm for a period of 30 days, using a mineral pulp density of 10% (*w*/*v*). Cell density was assessed every 24 h using a Neubauer dark-field chamber (Paul Marienfeld GmbH & Co. KG, Lauda-Königshofen, Germany) and a LEICA CME 1349521X optical microscope (40× objective, Leica Microsystems Inc., Buffalo, NY, USA) to monitor bacterial growth under each condition.

### 2.3. Isolation of Native Strains

The native bacterial strains present in the consortium were isolated using the serial dilution technique, using the medium in which the microbial consortium supported the highest microbial growth. A concentrated stock culture was subjected to tenfold dilutions. A volume of 100 μL from the 10^−6^ and 10^−7^ dilutions was plated using the surface-spreading method described by Puente-Flores [28]. The inoculated plates were incubated at 30 °C for 48 h. After incubation, the colonies were visually examined to determine their morphological characteristics, and representative colony types were selected for isolation. Each morphologically different strain was purified using repeated cross-streaking and incubated under the same conditions. The isolated strains were characterized based on their microscopic and macroscopic morphology; molecular identification was not performed, so their taxonomic classification is not included. The isolated strains were stored for further use [29].

### 2.4. Design of Bioleaching Experiments

Lithium bioleaching from the mineral concentrate was carried out using two approaches: a native microbial consortium and isolated native strains.

#### 2.4.1. Lithium Bioleaching by Native Microbial Consortium

Lithium recovery was evaluated using a Box–Behnken experimental design. Three factors were studied at three levels: stirring speed (0, 100, and 200 rpm), pulp density (10%, 20%, and 30%), and initial pH (6, 8, and 10). The response variables were lithium solubilization (%) and cell density (cells/mL). All treatments followed a one-step bioleaching approach, in which the mineral pulp was directly inoculated with a microbial consortium at 10% (*v*/*v*) in the selected culture medium. Each treatment was conducted in 250 mL baffled Erlenmeyer flasks. For the agitated treatments (100 and 200 rpm), the flasks were incubated in a LabTech LSI-3016A orbital shaker (LabTech, Anyang, Republic of Korea). The non-agitated treatments (0 rpm) were incubated statically in Felisa FE-29 incubators (Fabricantes Feligneo S.A. de C.V., Zapopan, Mexico). All bioleaching experiments were maintained at 30 °C for 18 days, a period selected to maintain favorable conditions for microbial growth and metabolic activity. All experimental runs were performed in triplicate, and the reported values correspond to the mean ± standard deviation.

After completion of the experimental design, numerical optimization was performed using Design-Expert^®^ version 13.0 software. The significance of the evaluated factors on lithium solubilization and cell density was determined by analysis of variance (ANOVA) at a significance level of *p* < 0.05. Numerical optimization was then carried out using the response surface superimposition method. The optimization criteria were defined as maximizing lithium solubilization and pulp density, minimizing agitation speed, and maintaining the initial pH and incubation time within the evaluated ranges.

#### 2.4.2. Bioleaching by Isolated Native Strains

Based on the optimal conditions identified in Section 2.4.1, bioleaching treatments were conducted, including a biotic control (BC), an abiotic control (AC), and each of the strains isolated from the microbial consortium. For the abiotic control and the isolated-strain treatments, the mineral concentrate was sterilized by autoclaving before incubation. The abiotic control was maintained without microbial inoculation, whereas each isolated strain was evaluated individually under identical experimental conditions. The bioleaching was performed by a one-step bioleaching approach in API sulfate culture medium. All treatments were incubated at 30 °C for 18 days under the optimized conditions determined in Section 2.4.1.

### 2.5. Lithium Solubilization Analysis

Lithium concentrations in the mineral samples were determined by atomic absorption spectrometry (AAS) following the procedure described by Ferreira et al. [30]. Analyses were performed using an Analyst 900Z spectrometer (PerkinElmer^®^, Shelton, CT, USA) equipped with a transversely heated graphite atomizer and a lithium hollow cathode lamp. Background correction was performed using the Zeeman effect to improve measurement accuracy.

Lithium solubilization (%) was calculated using Equation (1):

Lithium solubilization (%) = [(*Ci* − *Cr*)/*Ci*] × 100 (1)

where *Ci* is the initial lithium concentration in the mineral concentrate, and *Cr* is the residual lithium concentration in the solid residue after bioleaching.

### 2.6. X-Ray Diffraction Analysis

The effect of the bioleaching treatments on the mineralogical composition of the samples was evaluated by X-ray diffraction (XRD). Analyses were performed with a Rigaku Miniflex 600 diffractometer (Rigaku Corporation, Tokyo, Japan), operating in a 2θ angular range of 5–90° with a step size of 0.02°. Before analysis, the samples were ground and sieved to a particle size smaller than 200 mesh, homogenized, and mounted on the sample holder to ensure uniform exposure to the X-ray beam.

### 2.7. Comparison of Diffraction Patterns

Diffraction patterns were compared using Match! version 3.16 Build 283 software. First, a database containing the diffraction patterns obtained from the XRD analyses was generated. Subsequently, the diffraction pattern of the blank sample was incorporated and compared with the diffraction patterns of the samples obtained after bioleaching with each isolated native bacterial strain. This comparison allowed the similarities and differences between the diffraction patterns of the treated samples and the reference blank to be evaluated.

## 3. Results and Discussion

### 3.1. Comparison of Culture Media

**Figure 1:**
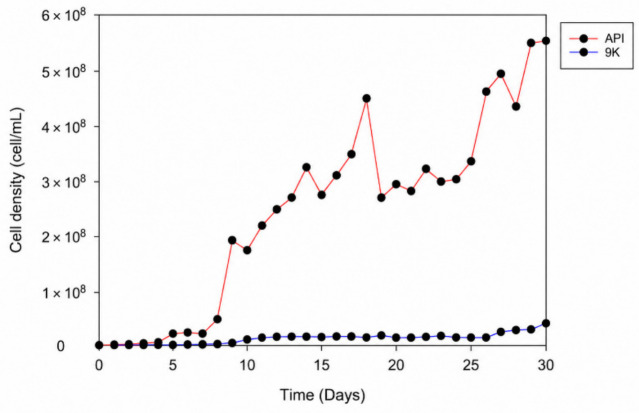
Cell density kinetics for sulfate API and 9K culture media. Rapid and exponential growth was observed in the sulfate API medium.

After 30 days of incubation, the API sulfate medium supported a substantially higher cell density (5.48 × 10^8^ cells/mL) than the 9K medium (3.97 × 10^7^ cells/mL) (Figure 1). Although the 9K medium was originally formulated for acidic environments, the addition of the mineral concentrate resulted in near-neutral pH values, whereas the API sulfate medium remained under slightly alkaline conditions throughout the experiment.

Previous studies have shown that environmental pH is an important factor influencing bacterial growth dynamics [31,32]. However, under the conditions evaluated in this study, pH did not appeared to be the main factor governing microbial growth, since both media maintained relatively stable pH values throughout the incubation period. Instead, the higher cell density observed in the API sulfate medium is more likely attributable to differences in medium composition. It has been reported that culture medium composition strongly influences microbial growth and metabolic performance, leading to variations in microbial activity and process efficiency depending on nutrient availability and medium formulation [33]. Therefore, the higher cell density achieved in the API sulfate medium suggests that its nutritional composition and physicochemical characteristics provided a more favorable environment for the development of the native microbial community than the 9K medium. Based on these results, the API sulfate medium was selected for all subsequent bioleaching experiments.

### 3.2. Isolation of Native Strains

Native microbial strains were isolated from the microbial using serial dilution followed by cross-streaking techniques. After incubating the diluted samples, three morphologically distinct colonies were identified and designated ITDB101, ITDR102, and ITDN103. These isolates were differentiated according to their colony morphology, following the criteria described in Bergey’s Manual of Systematic Bacteriology [34], including colony shape, elevation, margin, color, texture, and Gram-staining characteristics (Table 1). The purified isolates were subsequently preserved and used to evaluate their bioleaching performance.

The strains were named based on their pigmentation. Characteristics evaluated in solid medium after 48 h of incubation at 30 °C.

### 3.3. Evaluation of the Operational Parameters of Bioleaching by the Microbial Consortium

#### 3.3.1. pH

**Figure 2:**
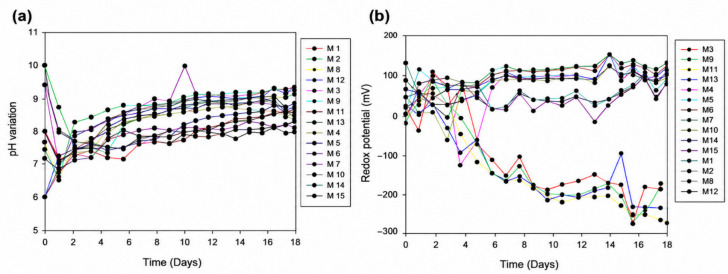
pH (**a**) and redox potential (**b**) behavior over time for the experimental treatments (M1–M13). The pH remained stable in most trials, while the Eh decreased in the tests without agitation (M2, M3, M8, M11, and M13).

During the 18-day bioleaching process, the pH of the culture medium showed a gradual increase all treatments. Although initial pH values were adjusted to 6, 8, or 10 according to the experimental design, most treatments converged to a final pH of 7.5 to 9.5, with stabilization occurring after approximately the third day of incubation (Figure 2a). In some treatments, an abrupt increase or decrease in pH was observed during the first 48 h, followed by a stable phase for the remainder of the experiment.

Despite these initial fluctuations, the final pH values were relatively similar among treatments, and no significant differences in lithium solubilization were detected among the evaluated pH levels (*p* > 0.05). Similar pH stabilization has been reported in other bioleaching systems, where microbial activity, together with the physicochemical properties of the culture medium, contributes to pH regulation [35].

#### 3.3.2. Redox Potential

During the 18-day bioleaching process, redox potential (Eh) values ranged from approximately +150 mV to −180 mV. Treatments M1–M13 correspond to the experimental conditions evaluated in this study. As shown in Figure 2b, the non-agitated treatments (M2, M3, M8, M11, and M13) developed more negative Eh values, frequently below −100 mV, whereas agitated treatments generally maintained values above +50 mV. This behavior reflects the lower oxygen availability under static conditions, which favors the establishment of a more reducing environment that may influence both microbial activity and mineral transformations.

According to Magaña & Rojas [36], negative redox potentials have been associated with the accumulation of ferrous ions (Fe^2+^) and reduced sulfur species, including thiosulfates and polythionates, generated during mineral reduction processes. Likewise, the black precipitate observed in the static treatments may be related to the formation of reduced iron- or sulfur-containing compounds under low redox conditions. Similar observations have been reported in anaerobic environments, where reducing conditions have been associated with hydrogen sulfide production and the activity of sulfate-reducing bacteria [36].

Although no direct relationship was observed between redox potential and lithium solubilization, these findings indicate that static conditions promoted a more reducing environment, which may influence the biochemical mechanisms involved in the bioleaching process. Similar effects of redox potential on metal mobility have also been reported by Moazzam et al. [16].

### 3.4. Cell Density Development During Bioleaching by the Microbial Consortium

**Figure 3:**
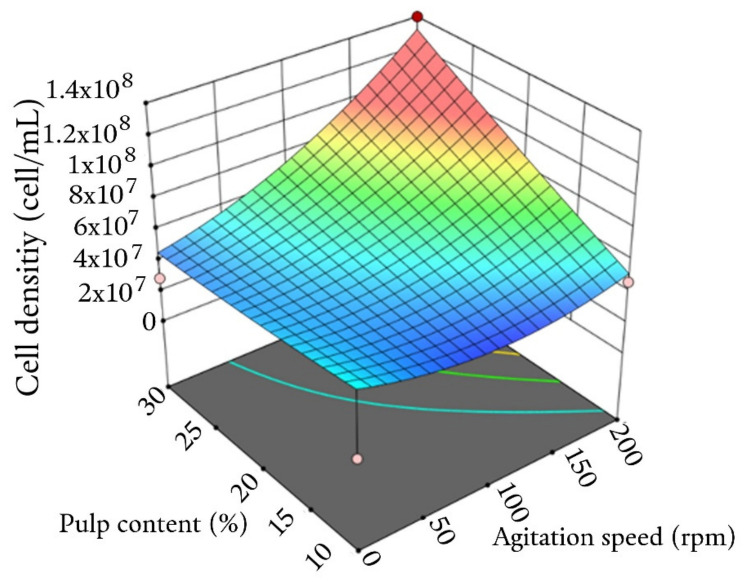
Response surface plot of the combined effect of pulp content (%) and agitation speed (rpm) on cell density (cell/mL).

According to the ANOVA results and response surface shown in Figure 3, both pulp content and agitation speed had a statistically significant effect on cell density (*p* < 0.05). Increasing agitation speed resulted in higher cell densities, most likely because of improved oxygen transfer, nutrient distribution, and heat dissipation within the culture medium [37]. These conditions favor the growth of planktonic bacteria, which exhibit greater metabolic activity in well-mixed suspension systems [20]. In contrast, under low-agitation or static conditions, microbial growth appeared to shift toward biofilm formation. Although this reduced the abundance of planktonic cells, it likely enhanced nutrient exchange at the solid–liquid interface [38,39]. This dual response reflects the adaptive strategies of the native microbial consortium under different hydrodynamic conditions and may explain the variation in cell density observed among treatments.

### 3.5. Lithium Solubilization by the Microbial Consortium

**Figure 4:**
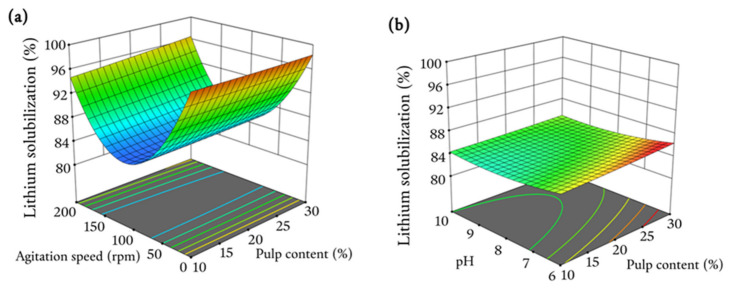
Three-dimensional response surface plots showing the effects of the evaluated factors on lithium solubilization. (**a**) Combined effect of agitation speed (rpm) and pulp content (%); and (**b**) combined effect of pH and pulp content (%).

The analysis of variance indicated that the regression model was statistically significant (*p* = 0.0023), with an R^2^ value of 0.972 and a coefficient of variation (CV) of 2.05%, demonstrating an excellent model fit. Among the evaluated factors, the quadratic term for agitation speed had a statistically significant effect on lithium solubilization (*p* < 0.0001). This effect is also evident in the response surface shown in Figure 4a, which reveals a non-linear relationship between agitation speed and lithium recovery. The highest lithium solubilization efficiencies (>90%) were obtained under both static conditions (0 rpm) and high agitation (200 rpm), whereas intermediate agitation (100 rpm) resulted in lower solubilization values (<85%). This behavior may be explained by the different metabolic strategies adopted by the microbial consortium under distinct hydrodynamic conditions. Under static conditions, biofilm formation may promote direct mineral solubilization through ion exchange processes and the production of hydrogen sulfide, facilitating mineral dissolution by biogenic acids [20,40]. In contrast, under high agitation, enhanced oxygen transfer may stimulate microbial activity under aerobic or microaerobic conditions, favoring indirect solubilization mechanisms involving the production of organic acids [41,42].

Regarding pulp density, the highest lithium recovery was obtained at a pulp density of 30%, followed by 10%, whereas 20% resulted in lower recovery efficiency (Table 2). The response surfaces presented in Figure 3 and Figure 4a further indicate that increasing pulp density promoted both microbial growth and lithium solubilization. This behavior may be attributed to the greater availability of mineral substrate, which provides additional nutrients and stimulates microbial activity [43]. However, excessive solid loading may also restrict oxygen diffusion and interfere with cell-mineral interactions, highlighting the importance of optimizing pulp density for efficient bioleaching.

In contrast, the initial pH did not have a statistically significant effect on lithium solubilization within the evaluated range (*p* > 0.05), as indicated by the ANOVA results (Table 2) and the relatively flat response surface shown in Figure 4b. Although all experiments were conducted under alkaline conditions, lithium recovery remained relatively stable across the evaluated pH values. This observation is consistent with the findings of Li et al. [44], who reported that lithium extraction using Na_3_PO_4_ increased as the pH shifted toward alkaline conditions and stabilized at approximately 80% when the pH exceeded 8. According to those authors, this behavior is associated with acidification reactions that promote the formation of LiH_2_PO_4_, a compound with greater water solubility, thereby increasing the concentration of dissolved Li^+^ in solution. Consequently, pH plays a critical role during the chemical precipitation stage, making the identification of an optimal pH essential for maximizing lithium recovery [45].

Although all experiments were conducted using an actual lithium-bearing mineral concentrate, the bioleaching performance observed in this study may vary when different mineral resources or waste matrices are evaluated. Differences in mineralogical composition, buffering capacity, and pH dynamics can influence bacterial growth and metabolic activity, ultimately affecting lithium recovery efficiency. Consequently, additional studies are needed to evaluate and optimize the proposed bioleaching process using a broader range of mineral and waste matrices.

### 3.6. Evaluation of Lithium Bioleaching Under Optimal Conditions by Isolated Native Strains

A numerical optimization was performed using the experimental data, and the model identified the following conditions as optimal: a pulp density of 30%, an initial pH of 6, static incubation (0 rpm), and an incubation period of 12 days at 30 °C. Based on the second-order response surface model, an empirical equation (coded factors) was generated to predict lithium solubilization (%), as shown in Equation (2).

Li solubilization (%) = 84.36 + 0.4350 × *A* + (−0.5775) × *B* + (−1.40) × *C* + (−0.1925) × *AB* + 0.4775 × *AC* + 0.9625 × *BC* + 0.0813 × *A*^2^ + 0.7513 × *B*^2^ + 12.58 × *C*^2^ (2)

where A represents pulp density (%), B is the initial pH, and C is the agitation speed (rpm).

The optimized conditions were subsequently validated under laboratory conditions to verify the model predictions. The optimization aimed to maximize lithium solubilization by maximizing pulp density, minimizing agitation speed, and maintaining the initial pH within the evaluated range. These operating conditions were applied to both the native microbial consortium and the isolated bacterial strains, and the corresponding results are presented in the following sections.

#### 3.6.1. pH Variation During Lithium Bioleaching by Isolated Native Strains

**Figure 5:**
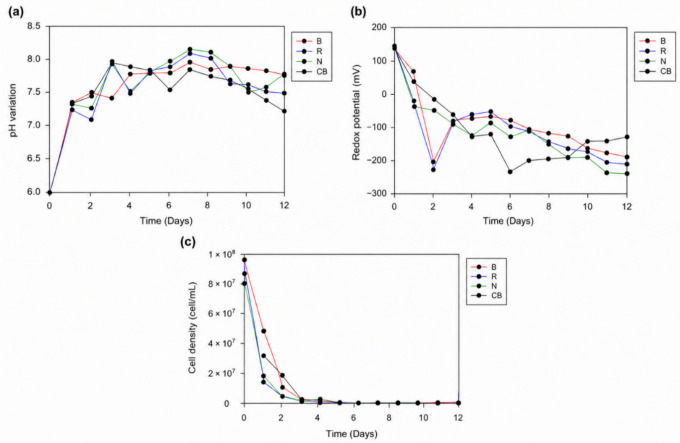
Kinetics of pH (**a**), redox potential (**b**), and cell density (**c**) for the optimal treatments, including the biotic control (BC) and the isolated strains: B (strain B), N (strain N), and R (strain R). The treatments were carried out at 30 °C, with a pulp content of 30%, and an initial pH of 6 without agitation.

Under the optimized conditions, the pH remained relatively stable, ranging from 7.0 to 8.2 despite an initial value of 6 (Figure 5a). This behavior is consistent with the findings of [46], who reported that sulfate-reducing bacteria (SRB) exhibit optimal metal-solubilizing activity under neutral pH conditions. Within this range, microbial growth is favored, and the reduction of sulfate to sulfide promotes the release of metal ions. The hydrogen sulfide (H_2_S) produced reacts with dissolved metals, contributing to both metal mobilization and a gradual increase in medium alkalinity. Consequently, pH plays a fundamental role in bioleaching by regulating both microbial metabolic activity and metal solubility [47].

#### 3.6.2. Redox Potential Behavior During Lithium Bioleaching by Isolated Native Strains

Under the optimized conditions, the redox potential remained within a negative range, consistent with the values observed in static treatments (Figure 5b). The absence of agitation likely limited oxygen transfer, creating reducing conditions that favor the activity of sulfate-reducing bacteria (SRB) and other anaerobic or microaerophilic microorganisms involved in metal solubilization [13]. Under these conditions, sulfate serves as the terminal electron acceptor during anaerobic respiration. Once transported into the cell, sulfate is activated by ATP sulfurylase, forming adenosine phosphosulfate (APS) and pyrophosphate [48]. APS, a high-energy intermediate, is subsequently reduced to sulfite, which is then converted into sulfide ions through metabolic intermediates such as metabisulfite, dithionite, trithionate, and thiosulfate [48,49]. These sulfide species contribute to the reductive dissolution of metal-bearing minerals by transferring electrons to insoluble oxides and promoting the formation of soluble metal complexes, while simultaneously increasing the alkalinity of the medium. The black precipitate observed during the experiment is consistent with the formation of ferrous sulfide (FeS), a product of microbial sulfate reduction under reducing conditions [50].

#### 3.6.3. Cell Density Development During Lithium Bioleaching by Isolated Native Strains

As shown in Figure 5c, cell density progressively decreased after the second day of incubation, coinciding with the onset of biofilm formation. This decline suggests a transition from planktonic growth to biofilm-associated cells, as microorganisms attached to the mineral surface and established biofilm communities. By the third day, the appearance of a black precipitate indicated the production of hydrogen sulfide (H_2_S), which reacted with iron compounds in the medium to form ferrous sulfide (FeS) [50]. Biofilm formation represents a critical stage in the bioleaching process because it enables microorganisms to establish microenvironments that promote the concentration and solubilization of metal ions through direct metabolic activity and physicochemical interactions with the mineral surface [51].

Although the observed bioleaching performance suggests the involvement of both direct and indirect mineral dissolution mechanisms, this study did not include the identification of intermediate metabolites or reaction byproducts generated during the process. Therefore, the proposed mechanisms are inferred from the experimental conditions and supported by previous studies reporting the participation of biofilms, organic acids, and sulfur-containing metabolites in mineral dissolution, as discussed throughout this section. Additional studies, including metabolite profiling and detailed mineralogical characterization of the residues, are needed to confirm the specific pathways responsible for lithium solubilization in this system.

Furthermore, the possible formation of microbial metabolites and the co-solubilization of other elements may influence the selectivity of lithium recovery and the efficiency of subsequent purification processes. Accordingly, further characterization of dissolved species and secondary metabolites would provide valuable insight into their potential effects on lithium recovery and purification.

### 3.7. Comparison of Lithium Bioleaching Between Isolated Native Strains and the Microbial Consortium

Under the optimal conditions predicted by the response surface model (30% pulp density, initial pH 6, static incubation at 0 rpm, and 12 days at 30 °C), the experimental validation confirmed a lithium solubilization of 94.5% by the native microbial consortium (Table 3), in close agreement with the value predicted by the model. This result validates the optimization strategy and demonstrates the predictive capability of the response surface model.

As shown in Table 3, all biotic treatments achieved lithium solubilization values exceeding 90%, whereas the abiotic control showed significantly lower recovery. The highest lithium solubilization was obtained with the native microbial consortium (biotic control), which outperformed the treatments inoculated with the individual bacterial strains (ITDB101, ITDR102, and ITDN103). These findings indicate that the native microbial consortium provided a more favorable environment for bioleaching than the isolated strains under the evaluated conditions. Native bacteria isolated from mineral-rich environments are generally well adapted to toxic and nutrient-limited conditions, enabling them to maintain metabolic activity and promote mineral dissolution under environmental stress [18]. Additionally, several studies have reported that mixed microbial cultures achieve greater metal recovery than pure strains, because of cooperative interactions, including substrate exchange, pH regulation, and complementary metabolic roles [18,52]. Collectively, these findings support the use of native microbial consortia as effective and environmentally sustainable strategy for lithium recovery.

### 3.8. X-Ray Diffraction Analysis

**Figure 6:**
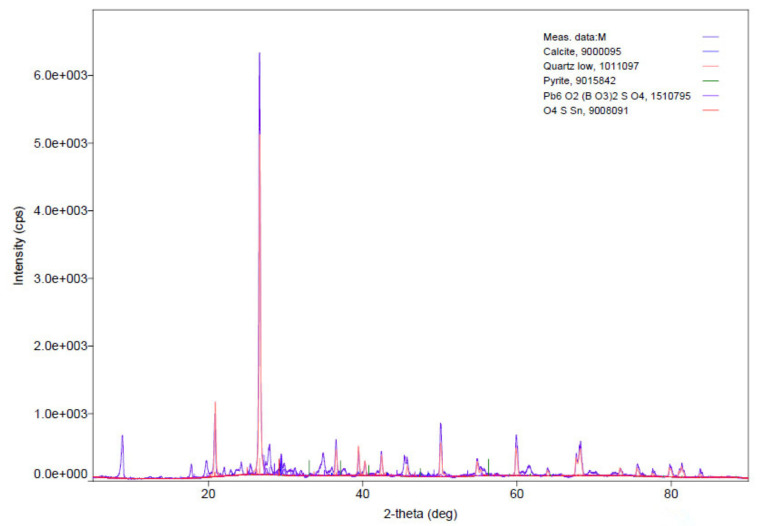
Diffraction pattern of the mineral concentrate before biolixiviation. Five major crystalline phases were identified: quartz (SiO_2_), calcite (CaCO_3_), tin oxide (SnO_2_), pyrite (FeS_2_), and Pb_6_O_2_(BO_3_)_2_SO_4_.

X-ray diffraction (XRD) analysis of the mineral concentrate identified five major crystalline phases: quartz (SiO_2_), calcite (CaCO_3_), tin oxide (SnO_2_), pyrite (FeS_2_), and Pb_6_O_2_(BO_3_)_2_SO_4_. The corresponding diffraction pattern is presented in Figure 6, which shows the characteristic diffraction peaks of the identified mineral phases. Quantitative phase analysis by the Reference Intensity Ratio (RIR) method indicated that quartz and calcite were the predominant crystalline components, accounting for approximately 92% and 5.4% of the total crystalline content, respectively.

### 3.9. Comparison of Diffraction Patterns

**Figure 7:**
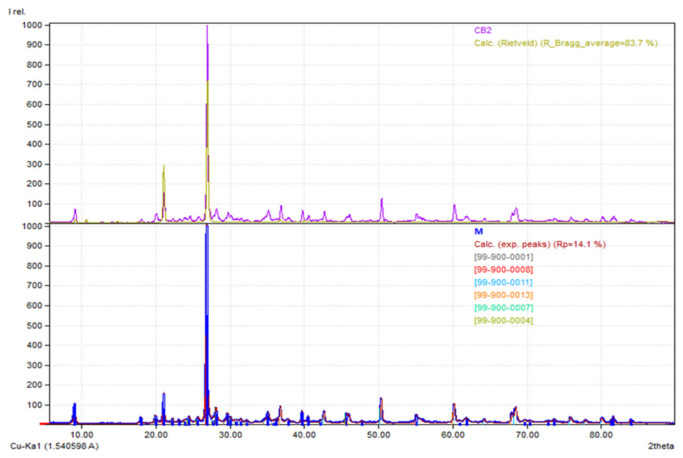
Comparison of the diffraction pattern between the blank and the BC sample (biotic control) with a convergence between phases of 83.7%.

Atomic absorption spectrometry (AAS) analysis of the mineral concentrate used in this study determined a lithium concentration of 0.0052%, which is well below the approximate detection limit of X-ray diffraction (XRD), estimated at approximately 0.5%. This explains the absence of diffraction peaks associated with lithium-bearing phases. According to information provided by the mining company and supported by previous mineralogical studies, the concentrate corresponds to a polylithionite-type clay, [KLi_2_Al(Si_4_O_10_)(F, OH)_2_] [25], in which lithium is structurally bound within a silicate matrix. Although lithium could not be detected directly by XRD, comparison of the diffraction patterns of the untreated sample (blank) and the optimized biotic treatment revealed clear differences in the mineralogical composition. As shown in Figure 7, both patterns exhibited a convergence index of 83.7%, indicating that measurable mineralogical changes occurred following the bioleaching treatment. These changes may reflect the partial dissolution or transformation of mineral phases resulting from microbial activity during the bioleaching process.

Despite the relatively low lithium concentration in the mineral concentrate, high lithium solubilization efficiencies were achieved during bioleaching. This behavior may be attributed to microorganism-mediated modification of the mineral surface through ion-exchange processes and the production of metabolites that promote mineral dissolution. These mechanisms may progressively destabilize the main mineral phases, exposing lithium-containing species associated with more abundant elements, including aluminum-bearing minerals [53]. However, since the dissolution of other metallic elements was not monitored, the selectivity of the process toward lithium could not be determined. Therefore, future studies should evaluate the simultaneous solubilization of additional elements to assess the selectivity of the bioleaching process and its implications for downstream lithium recovery.

## 4. Conclusions

The results of this study demonstrate the technical feasibility of recovering lithium from mineral concentrates through a one-step bioleaching process using a native bacterial consortium. The consortium, obtained through in situ enrichment and cultivation in API sulfate medium, achieved higher lithium solubilization than the individual bacterial strains. Under the optimized conditions (30% pulp density, an initial pH of 6, and static incubation), lithium solubilization exceeded 90%, with the highest recovery obtained using the complete native consortium. Throughout the process, the system stabilized under alkaline and reducing conditions that favored biofilm formation and the production of metabolites, including organic acids and sulfide species, which are known to promote mineral dissolution. X-ray diffraction analysis also revealed changes in the crystalline phases of the mineral matrix following bioleaching, supporting the occurrence of bioleaching-induced mineral transformations during the process. Overall, these findings demonstrate the potential of native microbial consortia as an effective and environmentally sustainable alternative for lithium recovery from mineral concentrates. This approach may be particularly valuable for the treatment of low-grade or mineralogically complex lithium resources, where conventional extraction methods are often less efficient. Further studies focused on metabolite characterization, process selectivity, and the recovery of co-solubilized elements will contribute to improving the understanding and practical application of this bioleaching strategy.

## Figures and Tables

**Table 1:** Colonial and cellular morphological characteristics of isolated bacterial strains.

Strain	Colony Form	Border	Transparency	Brightness	Colour	Texture	Elevation	Consistency	Cell Form	Grouping	Gram
B	Circular	Entire	Transparent	Brilliant	Non-pigmented	Smooth	Flat	Soft	Coco	No grouping	Negative
N	Irregular	Corrugated	Transparent	Brilliant	slightly orange	Rugged	Umbonate	Hard	Coco	No grouping	Negative
R	Irregular	Corrugated	Transparent	Brilliant	slightly pink	Rugged	Pulvinate	Hard	Coco	No grouping	Negative

**Table 2:** Effect of the pH, pulp density, and agitation on the cell density and lithium solubilization.

Experimental Run	pH	Pulp Density (%)	Agitation (rpm)	Lithium Solubilization (%)	Cell Density (Cell/mL)
1	8	10	200	95.00	4.84 × 10^7^
2	8	30	200	95.00	9.01 × 10^7^
3	8	30	0	98.08	1.85 × 10^7^
4	10	30	100	85.00	4.60 × 10^7^
5 *	8	20	100	83.85	4.74 × 10^7^
6 *	8	20	100	83.46	4.07 × 10^7^
7	6	10	100	85.00	2.35 × 10^7^
8	6	20	200	95.77	8.23 × 10^7^
9	10	20	0	97.69	2.01 × 10^7^
10	6	30	100	88.08	4.43 × 10^7^
11	6	20	0	99.23	2.55 × 10^7^
12	10	20	200	98.08	7.16 × 10^7^
13	8	10	0	100.00	1.17 × 10^7^
14	10	10	100	82.69	2.31 × 10^7^
15 *	8	20	100	85.77	4.87 × 10^7^

* Central points replicated in the Box–Behnken design (runs 5, 6, and 15). These treatments showed an average lithium solubilization of 84.36 ± 1.24% and a cell density of (4.56 ± 0.51) × 10^7^ cells/mL.

**Table 3:** Comparison of the percentage of Li solubilization for the optimal treatments with the native microbial consortium and isolated strains.

Condition	Lithium Solubilization (%)
ITDN103	91.92
ITDB101	93.27
ITDR102	91.73
Biotic control	96.35
Abiotic control	14.74

## Data Availability

The original contributions presented in this study are included in the article. Further inquiries can be directed to the corresponding authors.

## Abbreviations

The following abbreviations are used in this manuscript:

ITD	Instituto Tecnológico de Durango
EPS	Extracellular polymeric substances
API	Sulfate-Reducing Bacteria Broth
BC	Biotic control
AC	Abiotic control
AAS	Atomic absorption spectrometry
XRD	X-ray diffraction
Eh	Redox potential
M1-M13	Mineral treatments
Fe^2+^	Ferrous ions
H_2_S	Hydrogen sulfide
SRB	Sulfate-reducing bacteria
APS	Adenosine phosphosulfate
FeS	Ferrous sulfide
B	Strain B
N	Strain N
R	Strain R
SiO_2_	Quartz
CaCO_3_	Calcite
SnO_2_	Tin oxide
FeS_2_	Pyrite
RIR	Rietveld refinement
